# Advancing Lung Health in Ethiopia Through Research: A Success Story and A Pathway for the Future

**DOI:** 10.4314/ejhs.v35i1.1S

**Published:** 2025-12

**Authors:** Neil W Schluger, Rahal Argaw, Amsalu Bekele, Tewodros Haile, Joseph Huang, Eyob Kebede Etissa, Deborah A Haisch

**Affiliations:** 1 New York Medical College, Valhalla, New York, USA; 2 College of Health Science, Addis Ababa University, Addis Ababa, Ethiopia; 3 University of Colorado, Denver, Colorado, USA; 4 New York University Grossman School of Medicine, New York, USA; 5 Weill Cornell Medicine, New York, USA

The articles presented in this issue of the journal are the result of an extraordinary collaboration that began more than a decade ago among faculty at Tikur Anbessa Specialized Hospital, the Addis Ababa University School of Medicine, and pulmonary and critical care physicians from several medical schools and academic medical centers in the United States and Europe. This collaboration, known as the East African Training Initiative in Pulmonary Medicine (EATI), has fostered the development of the first generation of pulmonary physicians in Ethiopia.

EATI has led to the establishment of a robust infrastructure for clinical training in pulmonary and critical care medicine; supported the creation of the Ethiopian Thoracic Society (ETS)—now a major force for continuing medical education and national advocacy on lung health issues, including tobacco control and Ethiopia's response to the COVID-19 pandemic; and promoted research in pulmonary and critical care topics relevant to Ethiopian patients. Many physicians trained through EATI and now leading ETS have presented their work at major international scientific meetings and routinely participate in international research collaborations, training programs, and policy forums.

The articles published in this issue were solicited for a research symposium organized by the ETS and represent the collective efforts of a large group of clinicians, clinical researchers, mentors, faculty, and fellows. Together, these contributions mark a significant achievement and advance the shared goal of improving lung health in Ethiopia. Research moves the field forward by addressing critical questions that often begin with clinical observation: Why is my patient sick? Why is my patient not improving? The projects described in this issue represent essential first steps in answering these questions. To sustain and expand such efforts—and to build a durable research enterprise that supports lung health in Ethiopia—additional investment and strategic planning will be required.

All meaningful research begins with well-formulated questions that address common yet challenging clinical problems. Why is asthma so prevalent in Ethiopia? What are the causes of chronic lung disease in a population with, thus far, very low tobacco use? Is the incidence of lung cancer increasing? Which biomass fuels contribute most to indoor air pollution and lung disease? Which occupations pose the greatest risks to lung health? Can outcomes in critical illness be improved through simple, low-cost interventions? What are the most common causes of lung disease in children? Fortunately, Ethiopia now has a critical mass of highly trained pulmonary and critical care physicians with the expertise and insight needed to identify these questions and begin developing answers. What remains to be done?

**Research Training:** No one is born knowing how to conduct research. Just as physicians are trained to take clinical histories, perform physical examinations, understand respiratory physiology, conduct bronchoscopies, and interpret pulmonary function tests, they must also be trained in research methodology. This includes learning how to formulate a research question and hypothesis; design a rigorous study protocol; develop a statistical analysis plan and calculate appropriate sample sizes; ensure participant safety; interpret results accurately; and communicate findings clearly and effectively in peer-reviewed publications. These are skills that must be deliberately taught and learned.

Such training has been provided to EATI participants through several mechanisms, most notably the Methods in Clinical and Operational Research (MECOR) programs sponsored by the American Thoracic Society and the Pan-African Thoracic Society. As the number of trainees in pulmonary and critical care medicine has grown, however, so too has the need for sustained financial support to expand research training opportunities. Identifying funding sources capable of supporting rigorous research training—both within and outside Ethiopia—will be essential to maintaining the program's momentum. Potential sources include nongovernmental organizations, government agencies such as the Federal Ministry of Health, and international funders, including the European Union. Without such investment, sustaining these efforts will be difficult. Notably, over the past decade, several Ethiopian investigators have emerged as successful researchers; with adequate resources, much of this training could increasingly occur locally.

**Establishing the Physician-Investigator Career Path:** Research is not a hobby that can be pursued in spare moments; it is a demanding professional endeavor that requires protected time and institutional support. To sustain and expand the work described in this journal, Ethiopian medical schools and academic medical centers must create faculty positions in which a clearly defined portion of effort—whether 25%, 50%, or 75%—is dedicated to research. While these positions must ultimately attract external funding for salary support, it is equally important that clinical research be formally recognized and supported as a viable career pathway within academic institutions nationwide.

**Funding for Research Projects:** Although research need not be prohibitively expensive, it is never cost-free. Financial support is required at every stage of the research process. Investigators need protected time to conduct research, free from excessive clinical demands that can undermine scholarly productivity. Funding is also necessary to support key personnel, including statisticians, research assistants, and study coordinators, as well as to enable travel to international scientific meetings and, in some cases, publication in open-access journals.

Research funding is available from a range of sources, including the U.S. National Institutes of Health (NIH), the U.S. Agency for International Development (USAID), agencies within the European Union, and non-governmental organizations such as the Wellcome Trust and the Gates Foundation, among others. Competition for these funds is intense. Successful applications typically depend on strong partnerships, adequate time for proposal development, internal review, and administrative support, and often require multiple submissions before funding is secured. The articles in this issue present numerous promising ideas that could be developed into larger research projects and competitive funding proposals.

## Conclusions

Without research, meaningful progress in improving the lives of Ethiopians living with lung disease will not be possible. Implementation science to optimize the use of existing therapies, epidemiologic studies to clarify risk factors, and clinical research to evaluate new treatments for asthma, chronic obstructive pulmonary disease, and other chronic illnesses are all essential to improving patient care. The work presented in this issue of the journal is therefore a cause for celebration. It reflects the dedication and accomplishments of a broad community of physicians and partners committed to advancing lung health in Ethiopia.

These efforts have earned national and international recognition and have positioned Ethiopia's pulmonary and critical care community to deepen collaborations with global partners and expand the reach and impact of research. We have outlined key forms of support that will be necessary to sustain and grow these initiatives. We look forward to many more years of research productivity, innovation, and success from this remarkable community of clinicians and scientists.

